# Considerations for Regulatory Reusability of Dynamic Tools in the New Drug Development

**DOI:** 10.1007/s11095-025-03831-5

**Published:** 2025-03-04

**Authors:** Jiang Liu, Yuching Yang, Joga Gobburu, Cynthia J. Musante, Martin Klein, Liang Zhao, Rajanikanth Madabushi, Hao Zhu

**Affiliations:** 1 Office of Clinical Pharmacology (OCP), Office of Translational Sciences (OTS), Center for Drug Evaluation and Research (CDER), Food and Drug Administration (FDA), 10903 New Hampshire Ave, Silver Spring, MD 20993 USA; 2 University of Maryland Baltimore, Baltimore, MD USA; 3 Translational Clinical Sciences, Pfizer Research and Development, Cambridge, MA USA; 4 Division of Biometrics VIII, Office of Biostatistics, OTS, CDER, FDA, Silver Spring, MD USA; 5 Division of Quantitative Methods and Modeling, Office of Research and Standards (ORS), Office of Generic Drugs (OGD), CDER, FDA, Silver Spring, MD USA; 6 Quantitative Medicine Center of Excellence, CDER, FDA, Silver Spring, MD USA

**Keywords:** modeling, reusability, simulation, workshop report

## Abstract

Model-informed drug development (MIDD) approaches have become indispensable for new drug development and to address regulatory challenges. Dynamic tools, such as population pharmacokinetics (popPK), physiologically-based pharmacokinetics (PBPK), and quantitative systems pharmacology (QSP) models, are routinely employed to enhance the efficiency of drug development. Recently, the Fit-for-Purpose (FFP) initiative and the Model Master File (MMF) framework have emerged to support model reusability and sharing in regulatory settings. In this manuscript we share key insights from the Session "Pathways for Regulatory Acceptance of Dynamic Tools in the New Drug Space" of Workshop “Considerations and Potential Regulatory Applications for a Model Master File”, hosted by the U.S. Food and Drug Administration (FDA) and the Center for Research on Complex Generics (CRCG) and discuss the considerations for regulatory acceptance of dynamic modeling tools. Presentations at the workshop explored current practices in PBPK model evaluation, the potential for popPK models in bioequivalence (BE) assessments, and the implications of reusing models. Challenges such as context-specific validation, version control, and the impact of scientific and technological advancements on model reuse were emphasized. The workshop underscored the importance of clear regulatory pathways and structured frameworks for the consistent application of reusable models. The MMF's potential to streamline reviews and reduce redundancies was noted, although operational details require further elaboration. Continued collaboration among stakeholders is essential to refine model-sharing practices, enhance model validation processes, and promote transparency, ensuring that MIDD approaches remain robust and adaptable to evolving regulatory needs.

## Introduction

Model-informed drug development (MIDD) approaches have become a cornerstone in supporting new drug development. As a technical concept, MIDD involves creating and applying exposure-based, biological, and pharmacological models from preclinical and clinical data to address drug development or regulatory challenges. A variety of modeling and simulation tools, such as population pharmacokinetics (popPK), physiologically-based pharmacokinetics (PBPK), exposure–response, and disease modeling, are integral to MIDD. Recently, quantitative systems pharmacology (QSP) models and artificial intelligence/machine learning (AI/ML) models have been added to the MIDD toolbox. These tools are extensively used to streamline drug development and improve patient care. Regulatory agencies rely on MIDD approaches for decision-making and policy development, employing these tools for purpose such as to determine sample sizes, optimize dose selection, select endpoints or biomarkers, and identify target patients or subgroups. In regulatory settings, the applications of MIDD approaches include contributing to substantial evidence for new drug or drug product approvals, enabling extrapolation of efficacy findings across populations, informing risk–benefit assessments, and selecting appropriate regimens for general population and specific patient groups [1, 2].

While MIDD plays an integral role in modern drug development, building robust models requires significant resources and expertise, often following an iterative process updated with new data and knowledge. Enhancing model reusability where applicable can bring significant efficiencies to enable more routine application and evaluation. The Fit-for-Purpose (FFP) initiative offers a regulatory pathway for the acceptance of dynamic tools, also referred to as "reusable" models, in new drug development [3]. The Model Master File (MMF) framework recently introduced for complex generic drug development potentially also provides a sharable platform for intellectual property that is acceptable for regulatory purposes [4–6]. To facilitate resource and knowledge sharing and increase the efficiency/reusability of models in the new drug space and improve the community’s understanding on the FFP and MMF initiatives, the U.S. Food and Drug Administration (FDA) and the Center for Research on Complex Generics (CRCG) hosted a public discussion on “Pathways for Regulatory Acceptance of Dynamic Tools in the New Drug Space” in a hybrid public workshop titled " Considerations and Potential Regulatory Applications for a Model Master File (MMF)" on May 2 and 3, 2024 [7]. Speakers from the FDA, industry, and academia shared considerations in new drug development on: regulatory utility of the Fit-For-Purpose (FFP) Program to facilitate greater utilization of dynamic models in drug development programs; current practice in PBPK model evaluation and reusability; and potential of repeated usage of population PK models to support BE assessment. The scientific presentations were followed by a panel discussion on implementation issues of model sharing and reusability and the feasibility of MMFs in new drug development.

## Fit-For-Purpose Program

Model building in new drug development is typically an iterative process. For example, in dose selection, the clinical development program aims to reduce uncertainty around the final chosen dose. Although modeling tools translate nonclinical findings for dose selection, first-in-human studies often test a wide range of doses. As clinical trials progress and more data are collected, models are refined, leading to better predictions of the target dose range and more informed trial designs. Consequently, model structures, including covariate relationships and estimated parameter values, are continually updated as the clinical program advances.

However, some models with the same structure and parameter values may be reused across different drug development programs. As discussed by Dr. Hao Zhu, disease modeling is a prime example, where a single model can be applied to multiple programs. Model validation is generally guided by the principles of risk-based credibility assessment framework [9, 10]. This process begins with identifying the Question of Interest and Context of Use. The model influence, or the weight of model generated evidence in the totality of evidence, and the decision consequence, or the potential patient risk from incorrect decisions based on the totality of evidence, determine the model risk. High model risk typically necessitates additional validation activities and technical standards to ensure the quality of the model-generated evidence. The Context of Use is crucial in determining model risk and the required validation. While model influence and decision consequence are well-defined for models developed within specific programs, a "reusable" model should account for a wider range of scenarios. Consequently, the risk for a "reusable" model should be defined conservatively, covering a broad spectrum of potential outcomes. As a result, validation activities and technical standards may differ between a "reusable" model and one designed for a specific program.

The Fit-for-Purpose (FFP) program offers a regulatory pathway for the acceptance of dynamic tools, also referred to as "reusable" models, in drug development. This program is a collaborative effort between multidisciplinary review teams and external stakeholders in the development of modeling tools. To date, the FDA has granted "fit-for-purpose" designation to four applications. These include the Alzheimer’s disease model for clinical trial design developed by the Coalition Against Major Diseases (CAMD), the MCP-Mod tool for dose finding co-developed by Janssen Pharmaceuticals and Novartis Pharmaceuticals, the Bayesian Optimal Interval (BOIN) design for dose selection proposed by Dr. Yuan from the University of Texas and MD Anderson Cancer Center, and the Empirically Based Bayesian Emax Models for dose selection developed by Pfizer. The specific context of use, model evaluation, and conclusions for each program, as outlined in the determination letter, are detailed in Table I [3].

**Table I:** Summary of Context of Use, Model Evaluation, and Conclusion for FDA Granted “Fit for Purpose” Applications

Model	Context of Use	Review Assessment	Conclusion
Alzheimer’s Disease Model	Simulation tool to provide quantitative support in design and planning of clinical trials involving subjects with mild to moderate Alzheimer’s disease	1. Assumption, 2. Predictive Performance, 3. Development Platforms Note: The FDA commented that disease models are not intended to have a static constructure and characteristics. Model’s predictivity could be refined over time with additional knowledge, important covariates, mechanism characteristics of drugs. Plus, additional data should be incorporated	The model is scientifically supported and suitable for the purpose of aiding in design of future clinical trials in patients with mild to moderate Alzheimer’s disease
MCP-Mod	The goal of MCP-Mod is to serve as a principled strategy to explore and identify adequate doses for drug development. The MCP step aims to assess the dose–response signal using multiple comparison methods and to select the “best” model(s). The Mod step then entails fitting the selected model(s) to the data and estimating the target dose(s)	1. Use simulation studies to demonstrate the benefit of MCP-Mod compared to other approaches 2. Assess generality and applicability of the procedure 3. Evaluate publicly available software packages	Scientifically sound, determined fit-for-purpose in the outlined context
BOIN	To identify maximum tolerated dose (MTD) based on Phase 1 dose finding trials. MTD is defined as dose with dose limiting toxicity probability closet to a pre-specific target value	1. Methodology review focused on derivation of BOIN design 2. Identify the applicable scenarios (local BOIN design with non-informative prior) 3. Assess the simulation scenarios with the understanding of limitations 4. Software implementation of BOIN design	The review team finds that under the non-informative prior, the local BOIN design, in its revised form, can be designated fit-for-purpose
Bayesian Emax Model	Statistical methods and supporting software are proposed to improve the design and analysis of clinical trials whose primary purpose is to characterize the relationship between efficacy and dose to guide the dose selection for further development	1. Check assumption (e.g., identical treatment effect across multiple studies), applicability with comparable trial design (study population, randomization allocation, primary endpoint etc.) 2. Evaluate based on Goodness of Fit statistics (predictive probability for non-monotonicity) and its applicability 3. Apply through simulation studies and their generalizability 4. Evaluate Additional data source and R package	The review team finds that the proposed empirically-based Bayesian Emax model, including the goodness-of-fit (GOF) statistic, can be designated fit-for-purpose under the following conditions: (1) component studies for a new compound are homogeneous (2) the proposed GOF statistic is applicable (3) the model is identifiable (4) study-specific information is considered for dose selection

## Current Practice of PBPK Model Evaluation and Implications for Reusability

PBPK modeling and simulation is commonly employed to assess the impact of intrinsic factors (e.g., ontogeny, organ dysfunction, pharmacogenetics) and extrinsic factors (e.g., concomitant medications) on drug exposure to support safety and efficacy assessments in new drug development. As one major type of dynamic models, Dr. Yuching Yang discussed the FDA’s current practice in evaluation of PBPK models, emphasizing the importance of their validation and reusability based on consideration of factors such as the context of use (COU), the model risk, and its potential impact on regulatory decisions.

Developing a PBPK model requires substantial resources and integrates knowledge across multiple domains—biological, chemical, and pharmacological. Mechanistic representation is critical in PBPK model development, which translates this knowledge into a structured form, using the conceptual framework and mathematical representation. Once a base PBPK model is developed, it should undergo a validation process, typically with clinical data, to ensure it is robust enough to describe desired clinical scenarios. It is emphasized that, for regulatory review, the extent of validation depends on the model’s COU and the risk associated with the PBPK analysis.

**Fig. 1:**
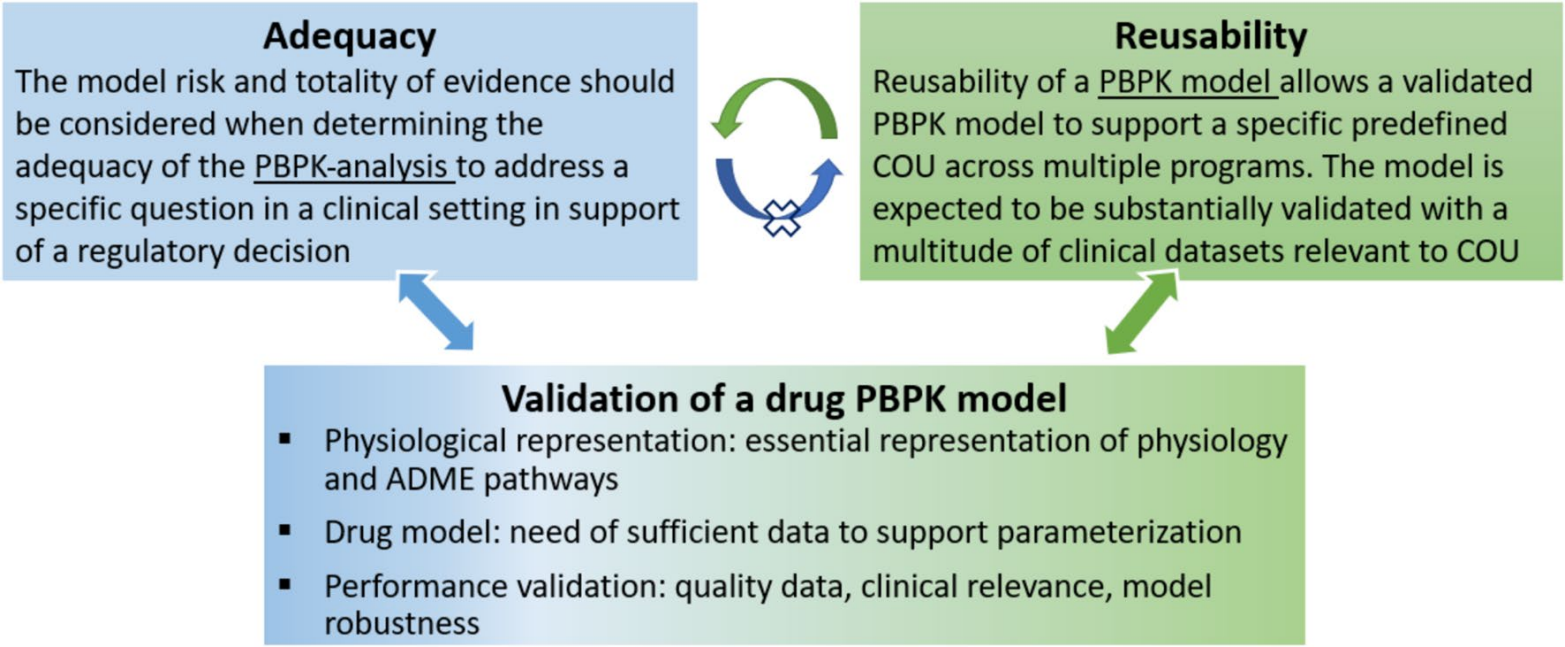
Adequacy of PBPK analysis *vs* reusability of PBPK models.

Dr. Yang explained the interplay between regulatory acceptance of a PBPK analysis and the potential for reusability of a PBPK model (as shown in Fig. 1). The adequacy of a PBPK analysis submitted for regulatory review is determined based on the model's risk and the totality of the evidence for addressing the specific question at hand. In contrast, a reusable PBPK model is expected to support a predefined COU across multiple programs. Reusability requires that the model’s assumptions and uncertainties are well-documented and that it is validated with clinical data relevant to the COU.

Therefore, acceptance of a PBPK analysis for regulatory decision-making does not automatically imply that the model and assumptions can be reused in future applications. A reusable model would need to be suitable for any analysis where the COU remains relevant. Reusability is based on a well-defined COU and a well-vetted development process. Over time, as models are refined and validated with clinical datasets, their reusability increases. Models developed using specialized PBPK software platforms are more likely to be reusable, as these platforms provide consistent model structures that ensure alignment in assumptions, system-dependent parameters, and mathematical representations.

Increasing clinical validation has led to the common practice of reusing previously validated PBPK models, especially in evaluating drug-drug interaction (DDI) liabilities for new drugs. In particular, models for sensitive substrates, inhibitors, or inducers of specific enzyme pathways are frequently reused. Detailed documentation of model development and validation is required for each PBPK model used, and the reusability of previously validated models is determined during the review process.

Dr. Yang also identified two key factors that may prompt the re-evaluation of a previously validated PBPK model’s reusability: changes in the model’s COU and advancements in science and technology. While these factors may appear straightforward, the practicalities of identifying such situations and determining the necessary actions remain underexplored. For instance, regulatory documentation on model reusability typically outlines accepted uses but rarely specifies when a model may no longer be appropriate. As a result, determining whether a previously approved model is suitable for new scenarios can require as much effort as evaluating a new model, regardless of past approvals.

There is also a need to balance the effort required to reassess model reusability following updates from new scientific insights (e.g., *in vitro* or *in vivo* data) or technological changes (e.g., software updates) to avoid redundant reviews, especially for models that have already been thoroughly evaluated. Redundant efforts can be minimized if the model was developed using specialized PBPK software and updates are clearly documented by the developers. However, a counterargument is that the PBPK software itself may need to be ‘verified’ and ‘validated’ before accepting updated models or documentation, which would require substantial resources.

Currently, OCP’s PBPK review focuses more on the adequacy of the submitted PBPK analysis in addressing specific clinical pharmacology questions rather than on evaluating the reusability of individual models, particularly for new molecular entities. However, reusing previously validated PBPK models is common in DDI assessments, especially when effects are assessed using certain sensitive substrates or exemplar inhibitors/inducers. These models typically have well-defined COUs, well-understood mechanisms, and have been repeatedly verified with robust clinical datasets.

## Potential of Repeated Usage of Population PK Models to Support BE Assessment

Pharmacokinetics (PK) modeling, particularly population PK (popPK) modeling, is increasingly utilized in drug development to assess bioequivalence (BE) across formulations. As discussed by Dr. Joga Gobburu, the potential for repeated usage of such models to streamline bioequivalence studies and new drug approvals has significant implications for the pharmaceutical industry. The concept of a MMF has emerged as a potential solution to eliminate redundant regulatory reviews, thereby optimizing the efficiency of the review process. However, its full implementation raises several critical issues around operational details and philosophical considerations that need clarification.

### Drug Master File and Bioequivalence Studies

The Drug Master File (DMF) is primarily designed to ensure the confidentiality of sensitive proprietary data, streamline the regulatory approval process, and provide greater flexibility and efficiency in submissions to regulatory authorities [8]. In essence, it serves as a repository for critical information that can be referenced by multiple applications, reducing the need for redundant submissions of the same data.

*In vivo* Bioavailability and Bioequivalence studies are essential for ensuring that the drug formulations used in clinical trials are comparable to the final marketed formulation. These studies play a vital role in bridging clinical trial formulations to the market, managing post-approval changes, and supporting lifecycle management for sponsors. The challenge lies in developing a framework that allows the repeated usage of population PK models to facilitate these bioequivalence assessments, particularly during formulation changes or regulatory submissions for new indications.

### Current Model Review Process

As discussed by Dr. Joga Gobburu, the current process for reviewing PK models, including popPK models, involves several steps and is iterative. Initially, the sponsor submits a model to the regulatory authority, which reviews it and then makes an approval decision. This model may later be used in a supplemental application for new purposes with newly available data, but it remains unclear whether regulatory authorities will always need to re-review the same model in its entirety during this secondary process. A key question he raised is whether changes in the review team, or new regulatory requirements, could lead to a different evaluation outcome for the same model.

The review process highlights the need for a more systematic approach that ensures efficiency without compromising the rigor of the review. Conducting a thorough analysis of these bottlenecks will be essential for proposing potential process improvements that can minimize unnecessary delays.

### Proposal for Model Master File (MMF)

One potential solution to avoid redundant reviews is the establishment of a MMF, which could allow sponsors to submit their PK models once, with the possibility of reusing them for multiple applications. This would eliminate the need for re-reviewing the same model or common aspects of the model each time a new application or supplemental application is filed.

However, Dr. Gobburu commented that the practicality of such a system may still be uncertain. For instance, while the MMF could save time, most models are typically updated with new data before submission as part of a supplemental application. If no updates are made to the original model, it raises the question of what the actual value of the original model for example in the assessment of Bioavailability and Bioequivalence of new drug formulations is.

Dr. Gobburu considers that the most significant potential usage of an MMF would likely be realized with complex models such as PBPK models or QSP models. Streamlining the review process for these models could lead to substantial time savings and increased regulatory efficiency.

### Philosophical Considerations and Challenges

Dr. Gobburu believes that one of the main philosophical questions surrounding the implementation of an MMF is whether regulatory authorities should review models in isolation from their specific application. A decision-oriented philosophy, which follows a decisions-information-analysis paradigm, asserts that the review of PK models cannot occur in a vacuum. The model would be evaluated in the context of the specific decision it supports, including its application in a particular drug development program.

In contrast, an academic approach to model development may advocate building a model first and then finding applications for it. However, from a regulatory perspective, an issue-driven approach is preferred. Regulators need to understand not just the technical aspects of the model but also its relevance and application to the specific drug or biologic under review. Pre-reviewing models without full knowledge of their application could lead to approval decisions that lack critical context, thus posing a regulatory risk.

### Operational and Implementation Considerations

There are several operational aspects of the MMF that need to be well-defined before such a system can be efficiently implemented to improve review efficiency. For instance, clear standards need to be established for the format of model files and the types of data that should be included in the report. Moreover, it will be necessary to determine whether the models should be agnostic to the software used in their development, ensuring that the MMF system remains flexible and accessible to sponsors regardless of their technological capabilities.

Another challenge arises when a model developed by one sponsor is used to support the application of another sponsor. For instance, in regulatory pathways such as 505(b)(2), 505(j), or biosimilars, a model developed by the original sponsor can, in theory, be reused by a second sponsor with regulatory approval. However, this requires the original sponsor to grant a right of reference to the second sponsor, which may not always be a straightforward decision. There may be competitive or proprietary concerns that inhibit such collaboration.

Moreover, in some instances, for the regulatory authority to approve the reuse of the model, the data driving the model’s development and validation may still need to be submitted and reviewed. As such, even with an MMF, some aspects of the review process may remain unchanged.

## Discussions

The FFP Initiative provides a pathway for regulatory acceptance of dynamic tools for use in new drug development. The FFP determination is made publicly available to facilitate greater utilization of these tools. However, due to the evolving nature of these types of drug development tools, models considered “fit-for-purpose” should be determined under a specific COU and assessed by appropriate model evaluation approach driven by COU and model risk. Even though a model is considered “fit-for-purpose,” the applicants can update the model or use other modeling approaches to address the same question of interest.

For PBPK applications, a fit-for-purpose approach based on COU is used to assess the adequacy of PBPK analyses (which typically include multiple models) in addressing specific clinical pharmacology questions, rather than focusing on the quality of an individual model across different applications. This is especially relevant for new molecular entities, where PBPK analysis may be applied at various stages for different COUs, with new data continuously emerging throughout development. The fit-for-purpose concept and the model credibility assessment framework [9, 10] provide flexibility to refine and reuse models without requiring additional intensive review activities typically expected for a major model modification program.

The workshop recognized that while MMFs have the potential to significantly streamline drug development particularly for complex generic drugs, there remain challenges related to the dynamic nature of models. The need for a structured approach to version control was highlighted, ensuring that updates to MMFs are properly documented and communicated to stakeholders. Additionally, it was suggested that a publicly available database of approved MMFs (e.g., following the approach of List of Drug Master Files maintained by the FDA [11]) could improve transparency and foster broader acceptance of reusable models​.

For the MMF application in the new drug space, the initial context of use of the models is expected to support bioequivalence evaluation in life-cycle management of NDAs (e.g., for formulation or manufacturing site change). The potential for repeated usage of population PK models to support bioequivalence assessments in new drug development offers substantial benefits, including reduced regulatory risk and greater efficiency. MMFs should provide detailed information on model development, qualification, and validation to support regulatory decisions. The model (including the parameters) is expected to be repeatedly used for the same drug substance and route of administration. Model structure and all parameters are robust and built through sufficiently large dataset in drug development programs aligned with current best biological and physiological understanding. There is sufficient information to evaluate model performance to the target context of use. Unlike the dynamic models from the FFP, for a MMF application change of the model structure or parameter values at a meaningful scale with new data should not be expected without regulatory re-assessment for the specific context of use initially accepted by the MMF. Although it was proposed that MMFs can be viewed as a type of electronic Drug Master File (DMF) submitted as an Electronic Common Technical Document (eCTD) [12], the practical implementation of a MMF (e.g., model sharing, transparency, and confidentiality for proprietary information protection) requires further clarification, particularly around operational details and philosophical concerns. Regulatory authorities will need to define the standards and application areas where MMF can add the most value to ensure that it benefits sponsors and facilitates the drug development process without compromising regulatory rigor.

## Conclusions and Future Perspectives

Dynamic tools and model sharing and reusability play an important role in modern drug development. The discussions revealed that model-based approaches like PBPK and PopPK, when validated for their context of use, can serve as regulatory assets by facilitating efficient drug development. The broader potential for reusing validated models across multiple programs including FFP and MMF present significant opportunities and complementary benefit for improving regulatory efficiency and decision-making.
